# Rapid and Sensitive Diagnosis of COVID-19 Using an Electricity-Free Self-Testing System

**DOI:** 10.3390/bios13020180

**Published:** 2023-01-23

**Authors:** Sheng Li, Wenlong Guo, Minmin Xiao, Yulin Chen, Xinyi Luo, Wenfei Xu, Jianhua Zhou, Jiasi Wang

**Affiliations:** 1 Guangdong Provincial Key Laboratory of Sensor Technology and Biomedical Instrument, School of Biomedical Engineering, Shenzhen Campus of Sun Yat-sen University, Shenzhen 518107, China; 2 Zhejiang Provincial Key Laboratory of Applied Enzymology, Yangtze Delta Region Institute of Tsinghua University, Jiaxing 314006, China; 3 School of Biomedical Engineering, Sun Yat-sen University, Guangzhou 510275, China

**Keywords:** SARS-CoV-2, self-testing, reverse transcription loop-mediated isothermal amplification

## Abstract

Rapid and sensitive detection of coronavirus disease 2019 (COVID-19) caused by severe acute respiratory syndrome coronavirus 2 (SARS-CoV-2) is essential for early diagnosis and effective treatment. Nucleic acid testing has been considered the gold standard method for the diagnosis of COVID-19 for its high sensitivity and specificity. However, the polymerase chain reaction (PCR)-based method in the central lab requires expensive equipment and well-trained personnel, which makes it difficult to be used in resource-limited settings. It highlights the need for a sensitive and simple assay that allows potential patients to detect SARS-CoV-2 by themselves. Here, we developed an electricity-free self-testing system based on reverse transcription loop-mediated isothermal amplification (RT-LAMP) that allows for rapid and accurate detection of SARS-CoV-2. Our system employs a heating bag as the heat source, and a 3D-printed box filled with phase change material (PCM) that successfully regulates the temperature for the RT-LAMP. The colorimetric method could be completed in 40 min and the results could be read out by the naked eye. A ratiometric measurement for exact readout was also incorporated to improve the detection accuracy of the system. This self-testing system is a promising tool for point-of-care testing (POCT) that enables rapid and sensitive diagnosis of SARS-CoV-2 in the real world and will improve the current COVID-19 screening efforts for control and mitigation of the pandemic.

## 1. Introduction

The coronavirus disease 2019 (COVID-19) pandemic caused a serious public health and economic crisis, that affected nearly half the world’s population and resulted in the death of more than 6 million people [1]. Accurate and effective diagnosis is extremely important to prevent the spread of severe acute respiratory syndrome coronavirus 2 (SARS-CoV-2) and help with the treatment [2,3,4]. To date, the most commonly used method for SARS-CoV-2 detection has been reverse-transcription polymerase chain reaction (RT-PCR), which is considered the gold standard because of its high specificity and sensitivity [5,6]. However, RT-PCR requires complex instruments with precise temperature control for thermal cycles. In addition, RT-PCR is always performed in laboratories and hospitals by trained operating professionals [7,8]. Therefore, it is unsuitable to be applied in point-of-care testing (POCT), which does not rely on sophisticated machines or trained professionals [9].

Isothermal amplification technologies, without the need for delicate equipment and professional personnel, are well-suited candidates for POCT and resource-limited settings (RLS) [10,11]. Loop-mediated isothermal amplification (LAMP) is an attractive method for POCT application because of its high specificity and the ability to amplify the signal rapidly at a constant temperature (60–65 °C), and provides diverse opportunities to monitor amplification results, from fluorescent to colorimetric dyes [12,13,14,15,16,17]. By combining the reverse transcription (RT), RT-LAMP can be completed for the detection of SARS-CoV-2 without the need for complicated instruments, which is favorable for the control of the COVID-19 epidemic [18,19]. Another issue for tests in the central lab is that people need to visit and line up at sample collection points, increasing the risk of further spreading the disease. Therefore, self-testing techniques allowing for an isolated strategy are of particular interest. Simplified isothermal approaches make it possible for individuals to perform self-checking at home and thus make significant contributions to the efforts against the COVID-19 pandemic [20]. Some products based on RT-LAMP have been approved by FDA for at-home testing [21], such as Lucira CHECK-IT COVID-19 Test Kit [22] and Cue COVID-19 test [23]. Usually, the LAMP reaction requires an incubator to maintain a fixed temperature, and the incubator typically consists of electric heaters and temperature controllers, which may represent a significant cost for self-testing [24]. Therefore, there is an urgent need for a rapid, easy-to-use, and cost-efficient at-home testing system for SARS-CoV-2 detection without the need for delicate equipment or well-trained personnel.

In this study, we developed an electricity-free, RT-LAMP-based color ratiometric analysis self-testing system to achieve a rapid diagnosis of COVID-19. This system employs a heating bag as the heating source and a 3D-printed box filled with phase change material (PCM) to incubate the RT-LAMP reaction, eliminating the requirement for a complicated electric heater.

## 2. Materials and Methods

### 2.1. Design of Self-Testing System

The 3D-printed box containing a test tube rack and a paraffin chamber was designed with Solid Works (Figure S1) and fabricated by a 3D printer using polylactic acid (PLA). The test tube rack is designed with 9 holes to hold a maximum of 9 test tubes. The paraffin chamber contains paraffin which undergoes a phase transition in the temperature range between 64 °C and 66 °C to regulate the incubation temperature. To improve thermal isolation, the external structure of the device employs a thick foam box made of polystyrene. The foam box is 28 cm in length, 18 cm in width, and 18 cm in height. The thickness of the foam box is 3 cm. To evaluate the thermal performance of the system, the 3D-printed box was designed with a 4 mm length × 4 mm width hole drilled in the top of the test tube rack, and the foam box has drilled a hole with the thermometer. The thermometer was inserted into the 3D-printed box through the drilled hole and sealed. The heating bags contain mixtures for an exothermic reaction, including CaO, magnesium alloy, and activated carbon. During the RT-LAMP reaction, the heating bag and water were put into the foam box to generate heat for the amplification reaction.

### 2.2. Simulation of Temperature Control Effect

The simulations were carried out by multi-physics simulation software (COMSOL 5.5). The size of the simulated box is the same as that in reality. The ambient temperature was set at 25 °C to simulate room temperature. The heat released from a certain mass of heating bags is fixed. We set the quantity of heat at 93 kJ by calculating the amount of heat given off by exothermic reactions. Water and heating bag combined result in a rapid exothermic reaction with a substantial release of energy and will heat the water in the foam box. We assume that the reaction is completed immediately when water seeped through the heating bag, and the temperature of the heating bag rises to the maximum. The quantity of the phase change material can be changed by adjusting the size of the 3D-printed box. In the simulations of this paper, the quantity of paraffin was set at 30 g. The box is sealed during the RT-LAMP reaction. Thus, the internal convection is simulated via isothermal domains. Heat flux to the surface is set to external natural convection to simulate the surface heat loss.

### 2.3. LAMP Reaction System

The LAMP reaction system contained 1 × Isothermal Amplification Buffer (New England Biolabs, Shanghai, China), 1.2 mM dNTP (Sangon Biotech, Guangzhou, China), 6 mM MgSO4 (New England Biolabs, Shanghai, China), 320 U mL^−1^ Bst 2.0 DNA polymerase (New England Biolabs, Shanghai, China), 160 U mL^−1^ Bst 3.0 (New England Biolabs, Shanghai, China). Primers used for the LAMP are shown in Table S2. A primer mix consisting of 1.6 μM of FIP and BIP, 0.2 μM of F3 and B3, and 0.4 μM of LF and LB was added to the reaction. RT-LAMP reactions were carried out in 0.2 mL PCR tubes. After adding the 1 μL sample, the final volume of the RT-LAMP reaction was 20 μL. In addition to the above reagents, the real-time LAMP reaction mixture contains 1 × LAMP Fluorescent Dye (New England Biolabs, Shanghai, China). The LAMP mixture was incubated at 65 °C for 50 min in a LongGene PCR System and the fluorescent signal was recorded every 1 min. The reaction mixture used in the system contains hydroxy naphthol blue (Aladdin, Shanghai, China).

### 2.4. Image Processing

After the amplification reaction, pictures of the test tubes were taken on a white background with a cellphone. Images acquired with the cellphone were processed using Matlab. The process is as follows: (i) Color channels red (R), green(G), and blue (B) of the original image were split. (ii) The value of G channel was divided by that of the R channel to obtain the G/R ratio. Then, we used the G/R ratio to discriminate the samples because the G/R ratio has the highest signal-to-noise ratio. (iii) The binary image was generated after a threshold correction. After image processing, the positive samples (originally blue) are white and the negative samples (originally purple) are black, and only the positive sample can be visualized.

### 2.5. Cell RNA Extraction

The total RNA was extracted from the 293T cells that were transfected with the pEF plasmid containing the N gene of SARS-CoV-2 to simulate real samples. The transfected cells were incubated in Dulbecco’s modified Eagle’s medium (DMEM) with 10% fetal bovine serum, 0.1 mg streptomycin, and 100 units mL^−1^ penicillin for 48 h. Then, the RNA was extracted by using GeneJET RNA Purification Kit. The concentration of RNA was measured by using the NanoDrop (Thermo Fisher Scientific, Waltham, MA, USA).

## 3. Results

### 3.1. Self-Testing System Based on RT-LAMP

**Figure 1:**
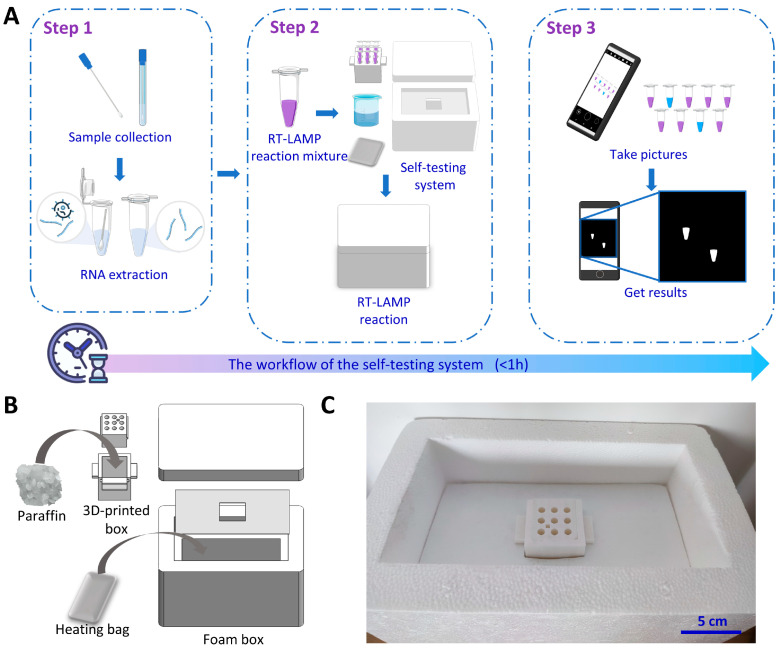
Detection of SARS-CoV-2 with the self-testing system. (**A**) Workflow of detection of SARS-CoV-2 with the self-testing system. The detection process using this self-testing system includes three steps: RNA preparation, RT-LAMP reaction, and data processing. (**B**) Schematic of incubation system. The system consists of a foam box, a heating bag, and a 3D-printed box. (**C**) Photograph of the incubation system.

Figure 1A shows the workflow of detection of SARS-CoV-2 using our self-testing system. It includes three steps: (1) The samples are self-collected and the viral RNA is extracted from the sample. (2) The extracted RNA is subjected to the RT-LAMP reaction using the self-testing system for temperature control. (3) The visual detection results could be read by the naked eye or by applying ratiometric processing to the images from a cellphone. The self-testing system employs a polystyrene foam box, a 3D-printed box, and a heating bag as the incubation system (Figure 1B,C). The incubation system consists of 2 parts, one containing a heating bag and a foam box as the heater case, and the other containing a 3D-printed box with paraffin. The addition of water to the heating bag triggers the exothermic reaction. A polystyrene foam box was employed for thermal insulation, which is not only cost-effective but also convenient for the storage and transportation of test reagents. The detection tubes are placed in the 3D-printed box filled with paraffin at the appropriate phase transition temperature for RT-LAMP. Paraffin has a large specific heat capacity and low thermal conductivity. The addition of paraffin absorbs the heat released by the heating bag. After the system has reached the paraffin’s phase transition temperature, additional energy generated by the reaction is consumed for phase transition, rather than increasing the temperature of the system. When the exothermic reaction has been consumed and the system starts to cool down, the paraffin prolongs the time of the constant temperature by releasing latent heat. Therefore, the reaction tubes could be kept at a constant temperature to complete the RT-LAMP, enabling electricity-free RNA detection.

### 3.2. Verification and Optimization of the Temperature Control Effect in Self-Testing System

**Figure 2:**
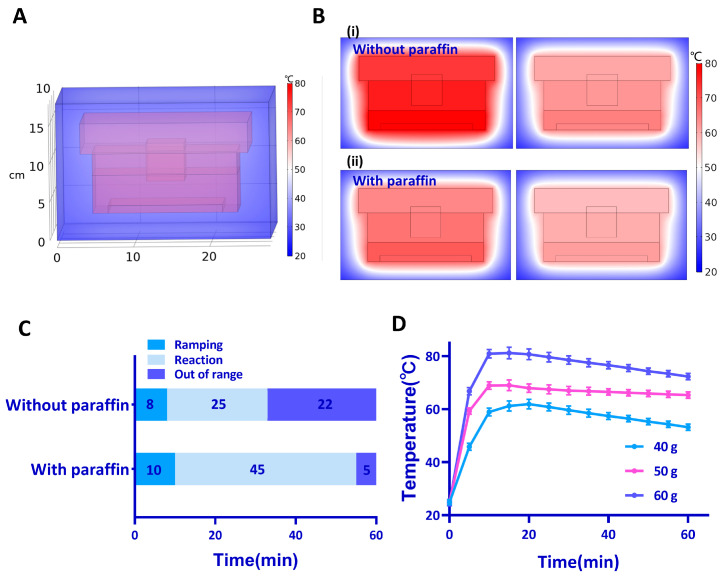
Temperature control effect of the self-testing system. (**A**) Simulations of temperature distribution inside the foam box. (**B**) Simulations of temperature distribution (**i**) without and (**ii**) with the paraffin. The left column shows the temperature distribution when the temperature inside the 3-D printed box is at maximum, and the right column shows the temperature distribution at 60 min. (**C**) Different times in the absence and presence of paraffin for temperature ramping, reaction, and out-of-range. (**D**) The temperature inside the 3D-printed box with different heating bags.

To ensure the amplification efficiency of RT-LAMP, we evaluated and optimized the temperature control effect of the self-testing system. First, the temperature inside the self-testing system was simulated by COMSOL5.5 to evaluate the temperature control effect in different conditions. The ambient temperature was set at 25 °C to simulate the room temperature. The results showed that the temperature distribution inside the foam box was even (Figure 2A,B). However, in the absence of paraffin, the temperature reached about 80 °C at first, which was over the temperature for RT-LAMP. Temperatures above 70 °C may inhibit the activity of the polymerase enzymes which will slow down the reaction. The paraffin lowered the maximum temperature of the system by absorbing excess heat as latent heat and prolonged the time of the constant temperature by releasing latent heat to keep the suitable temperature for the RT-LAMP reaction.

To verify these simulations, the temperature control effect of this system was tested under different conditions. We originally used CaO to provide heat. Once water is added to the heater case, the water seeps through and reacts with CaO. However, the temperature decreased quickly after reaching climax. To maintain a suitable temperature, independent of ambient conditions, we used paraffin which undergoes a phase transition in the temperature range between 64 °C and 66 °C to maintain the desired temperatures range for RT-LAMP. In the presence of paraffin, the time in target temperature was increased by 20 min (Figure 2C and Figure S2). We further explored the temperature control effect of the different masses of paraffin (Figure S3). The 3D-printed box filled with 25 g paraffin maintained about 50 min at the appropriate temperature. In summary, the addition of paraffin extended the time to keep at a fixed temperature between 60 and 65 °C, which is the required temperature range for the RT-LAMP reaction.

A large amount of CaO (200 g) was needed to reach the appropriate temperature, thus the heating method with CaO was inefficient. To obtain a better heating efficiency, we attempted to use the heating bags containing mixtures for an exothermic reaction, including CaO, magnesium alloy, and activated carbon. Different heating bags release different amounts of heat, which allows the system to be maintained at different temperatures (Figure 2D). The temperature of this system was higher when heavier heating bags were used. Redundant heating bag mass released a substantial amount of energy that exceeded the heat required for phase change, increasing the system’s temperature over the phase transition temperature. In all the experiments described later, we used a 50 g heating bag as it enabled incubation temperatures between 60 and 65 °C, which is the suitable temperature range for the RT-LAMP reaction. The heating bag prolonged the time interval at the fixed temperature and decreased the temperature ramp up time.

We also tested different volumes of water (Figure S4C). Excess water lowered the incubation temperature and extended the temperature ramp up time, which was consistent with the simulations (Figure S4A,B). Finally, 700 mL water was used for the following experiments. After optimization, the temperature could be kept at 60–65 °C for more than 60 min, which is long enough for the RT-LAMP reaction.

### 3.3. Feasibility of the Self-Testing System

**Figure 3:**
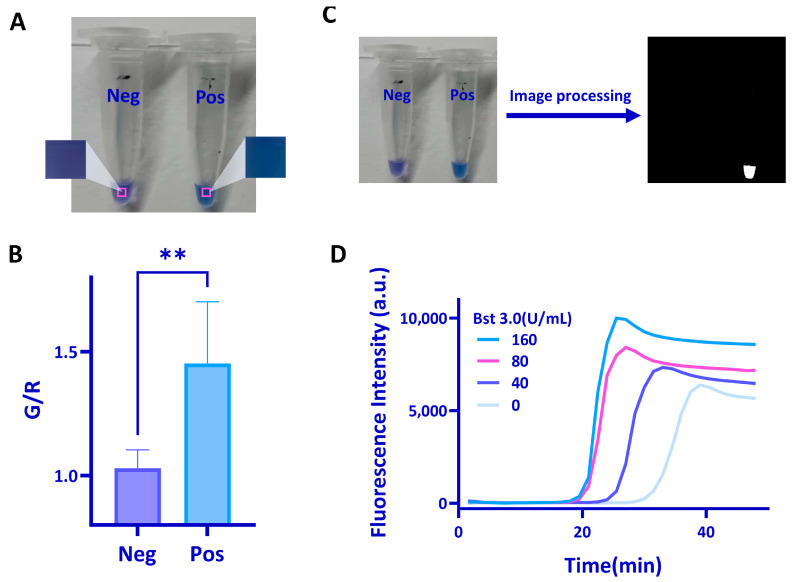
Feasibility test of the system. (**A**) Image analysis of the positive and negative samples. (**B**) G/R ratio of negative and positive samples calculated from images taken with a cellphone. The statistical analyses were performed using *t*-test, two-tailed, where ns = not significant with *p* > 0.05, the ** denotes significant differences with *p* values between 0.0001 to 0.001. (**C**) The images before and after G/R processing. (**D**) Real-time amplification curve of RT-LAMP with different concentrations of Bst3.0.

To verify the feasibility of the system, we next tested the LAMP reaction by using the SARS-CoV2 N gene. The LAMP results could be determined by the naked eye. The hydroxy naphthol blue (HNB), an Mg^2+^ indicator premixing in the LAMP reaction solution, was used here to provide colorimetric readouts. The one-pot reaction also reduces contamination risks since the assay does not need to open the reaction tubes for detection of LAMP products [25]. Typical colorimetric methods for detecting nucleic acid amplification usually evaluate absolute changes in color intensity. However, these colorimetric methods are usually difficult to distinguish similar colors, especially when the concentration of the template is low [26]. We here employed ratiometric measurements, which compared two independent measurements made under the same conditions, to improve the robustness of visual readouts. First, we analyzed the images of the negative and positive samples taken with a cellphone (Figure 3A). Readout images were split into three color channels (red, green, and blue, RGB), and the values of each channel were analyzed (Figure S5). The values of the R channel had the greatest difference between positive and negative outcomes. By applying the R channel to analyze, there are four possible combinations for ratiometric analysis: G/R, R/G, B/R, and R/B (Figure S6). We found the G/R ratio had the highest signal-to-noise ratio, and there was a significant difference between negative and positive results (Figure 3B). As a result, we chose the G/R ratio for the following experiments. With further image processing (Figure S7), the results could be converted to a yes/no binary outcome and reduced the impact caused by the unclear color change (Figure 3C).

Next, we evaluated the quantitative detection capability of this system by using the N genes at various dilutions. Through the ratiometric measurement, the original images of the test result were converted into a mono-colored image, and the sample with the concentration of 400 copies/μL could be identified (Figure S8). To further reduce the reaction time, we used Bst3.0 to accelerate the LAMP reaction. The results showed that the addition of Bst3.0 improved the amplification efficiency (Figure 3D). The amplification reaction can be completed within 30 min after optimization. It took about 10 min to reach 65 °C by using this system. Therefore, the detection could be completed within 40 min.

### 3.4. Evaluation of the Self-Testing System

**Figure 4:**
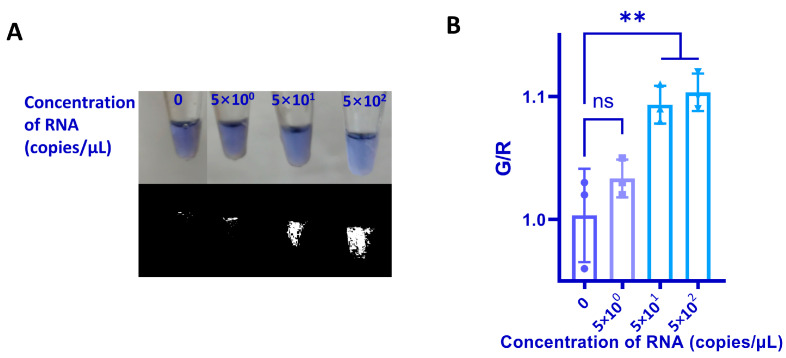
Sensitivity evaluation of the self-testing system. (**A**) Visual outcome of a 10 times dilution of SARS-CoV-2 RNA positive control ranging from 500 to 5 copies/μL. The top image is the original image taken with a cellphone. The bottom image is the binary image after G/R processing. (**B**) G/R ratio of the RT-LAMP reaction with different concentrations of SARS-CoV-2 RNA. G/R ratio of each concentration was calculated from three parallel tests and represent the mean value of the three tests. The statistical analyses were performed using a one-way analysis of variance, where ns = not significant with *p* > 0.05, and the ** denotes significant differences with *p* values between 0.0001 to 0.001.

Under the optimized experimental conditions (Table S3), reference RNA of SARS-CoV-2 with a series of dilutions ranging from 500 copies/μL to 5 copies/μL was used to determine the LOD of the self-testing system for SARS-CoV-2 detection. The results were analyzed by ratiometric measurement (Figure 4A,B). It is noteworthy that it is difficult to distinguish the color change between the negative sample and the positive samples, but the G/R ratio can help to distinguish the signal significantly. With our self-testing system, 50 copies/μL of target RNA could be detected, which can cover the majority of COVID-19 positive samples (median ~10^2^–10^3^ viral copies/μL [27]). This system can detect virtually all infectious individuals and can provide accurate results to users, which is applicable to the daily diagnosis of COVID-19.

### 3.5. Detection of SARS-CoV-2 in Transfected Cells Using the Self-Testing System

**Figure 5:**
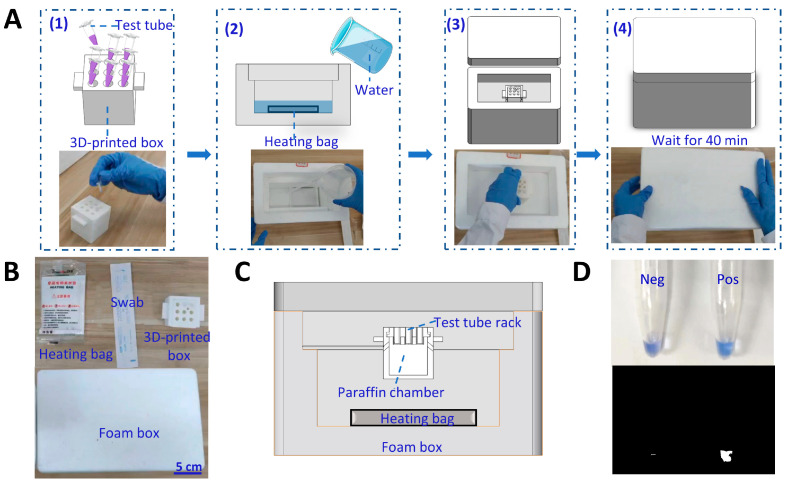
Test protocol and the design of the self-testing system for POCT. (**A**) The self-testing system scheme for performing the detection of SARS-CoV-2. (**B**) Illustrations of the self-testing system. (**C**) Explored view of the self-testing system. (**D**) The results of the RNA sample extracted from the SARS-CoV-2 transfected cells by using the self-testing system.

Finally, to demonstrate the practical detection capability of our system, we used the self-testing system to detect RNA in simulated samples. The RNA was extracted from SARS-CoV-2 transfected cells and was employed to simulate the real sample. The self-testing system was then tested for potential as a home testing platform. The detailed detection steps are shown in the scheme (Figure 5A). First, after self-collection and RNA extraction, the RT-LAMP amplification reaction mixture is prepared by the addition of the extracted RNA, and the test tubes with the mixture are placed into the 3D-printed box. Next, the heating bag and water were put into the foam box to start the exothermic reaction, and the 3D-printed box was put inside the box for the RT-LAMP reaction. Then, the box was covered by the lid and waited for 40 min. After the RT-LAMP, results should be read out or a picture taken immediately. This system is electricity-free and cost-efficient (Figure 5B,C), and can provide accurate results to end-users. The color of positive samples changes from violet to sky blue while the negative samples do not change. Results can be recognized by the naked eye or by taking pictures with a cell phone for more accurate results via G/R processing (Figure 5D). The total cost is less than $2 per test (Table S1). The results demonstrate the potential to use the self-testing system as a convenient platform for scanning individuals at home.

## 4. Discussion

In this study, we developed an electricity-free, cost-effective self-testing system without permanent instruments for SARS-CoV-2 detection at the POCT. The temperature control system is capable of maintaining the reaction temperature for more than 60 min. This system can identify down to 50 copies/μL of the SARS-CoV-2 RNA, which is sensitive enough to detect SARS-CoV-2 RNA in clinical samples. Our self-testing system offers several advantages:

(i) This is a cost-efficient and instrument-free platform. By using chemical heater reactions and paraffin, effective temperature control is achieved for RT-LAMP reaction without the need for electronic equipment which may represent a significant cost reduction for home testing. Compared to Lucira COVID-19 Test kit (Table S4), the total cost of our system is no more than $2 per test, which is 18 times less expensive than a COVID-19 Test kit. The heater case, including the 3D-printed box and the foam box, can be reused. The heating bag is similar to the heat source used in Meals Ready to Eat, thus it does not produce any chemical waste. Our self-testing system is made of readily available and low-cost materials without the need for electric heaters and sophisticated readers. The cost of our system could be further reduced by optimizing the reaction reagents and the 3D-printed box. The system needs about 10 min to reach 65 °C which makes the system have a longer time-to-result compared to other FDA-authorized molecular POC tests. Although the exothermic reaction can be completed immediately, it takes some time for the water to be heated because we used a big heater case and a large amount of water. The size of the heater case and the amount of water could be further optimized to accelerate the heating process. Since the rack can hold a maximum of 9 test tubes, our system can detect a maximum of 9 samples per operation (the cost will increase by $0.83 for each additional sample). The throughput could be further increased by redesigning the tube rack. In contrast, the Lucira COVID-19 Test kit can only detect one sample in one kit.
(ii) This is a rapid and accurate diagnosis. Lateral flow test can provide simple and rapid detection, but its sensitivity and specificity are relatively low because it relies on immunoassay technology [20]. The current RT-PCR method in centralized laboratories involves a significant long turnaround time between sample collection and results. Our system can detect SARS-CoV-2 in 40 min, which is faster than COVID-19 RT-PCR methods in terms of turnaround time. We used reference RNA of SARS-CoV-2 to determine the LOD of the self-testing system, and 50 copies/μL of target RNA could be detected which is sensitive enough to detect SARS-CoV-2 RNA in the majority of COVID-19 positive samples (~10^2^–10^3^ copies/μL on average). The ratiometric measurement is used and the result images could be converted to a yes/no binary outcome, which improves the detection accuracy of the system.
(iii) This is a contamination-free nucleic acid-based self-testing system. Our self-testing system combines visual detection in a single, closed test tube, reducing the contamination risk and simplifying the test operation. Strategies based on self-checking also minimize the risk of cross-infection and continuous spread. Given some RNA extraction-free methods have been developed for RT-LAMP to detect SARS-CoV-2, we will further develop a “sample-to-answer” diagnostic platform.

It should be admitted that no clinical samples are really tested to evaluate the selectivity and sensitivity of the self-testing system. The applicability and robustness that combines the system with POC nucleic acid extraction technologies has not been verified. Therefore, the performance of this system in practical application needs to be validated in future work by using clinical samples in POCT.

## 5. Conclusions

In summary, an electricity-free, RT-LAMP-based color ratiometric analysis self-testing system was developed for the detection of SARS-CoV-2. The cost-effective self-testing system shows a superior detection capability and is expected to become a powerful tool for control of the current COVID-19 pandemic. Our self-testing system could be used in daily tests at home, and will help to prevent the cross-infection and continuous spread of infectious diseases occurring in public places. In addition, with suitable modifications of the primer and probe, this system can also be used for the detection of other pathogens or viruses, particularly in countries without reliable electric power. We envision this self-testing system could have significant practical value for controlling and mitigating the pandemic.

## Data Availability

Not applicable.

## Supplementary Materials

The following supporting information can be downloaded at: https://www.mdpi.com/article/10.3390/bios13020180/s1, Figure S1: Design of the 3D printing box; Figure S2: The temperature in the 3D-printed box with and without paraffin; Figure S3: The temperature in the 3D-printed box with different masses of paraffin; Figure S4: The temperature control effect with different amounts of water; Figure S5: The RGB values of negative and positive samples; Figure S6: Analysis of the four possible RGB ratiometric combinations; Figure S7: Each step of the G/R ratiometric approach for the self-testing system; Figure S8: Visual readout of different concentrations of the plasmid; Table S1: Calculation of the cost of the self-testing system; Table S2: Reference primers were used in the study; Table S3: Optimized conditions of the self-testing system; Table S4: Comparison with Lucira COVID-19 Test kit.
