# Supplementary material for: Rapid and Sensitive Diagnosis of COVID-19 Using an Electricity-Free Self-Testing System
Source: Biosensors (Basel). 2023 Jan 23;13(2):180. doi: 10.3390/bios13020180 (PMC9953845; doi:10.3390/bios13020180)
Supplement: Supplementary file 1 [file biosensors-13-00180-s001.zip › biosensors-2129322-supplementary.pdf]

biosensors

MDPI

Supporting Information

Rapid and Sensitive Diagnosis of COVID-19 Using an Electricity-Free Self-Testing System

Sheng Li [1,†], Wenlong Guo [1,†], Minmin Xiao [1], Yulin Chen [1], Xinyi Luo [1], Wenfei Xu [2] Jianhua Zhou [1,3,*] and Jiasi Wang [1,3,*]

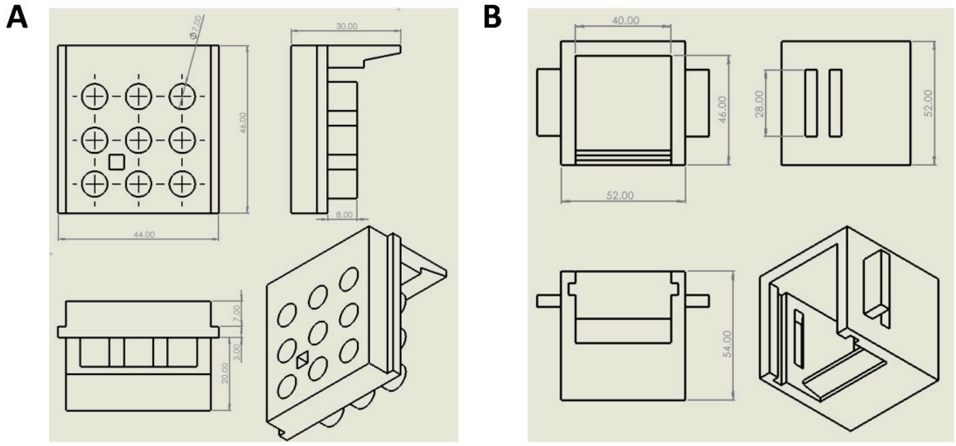

Figure S1. Design of the 3D-printed box. A) Engineering drawing of test tube rack. B) Engineering drawing of paraffin chamber.

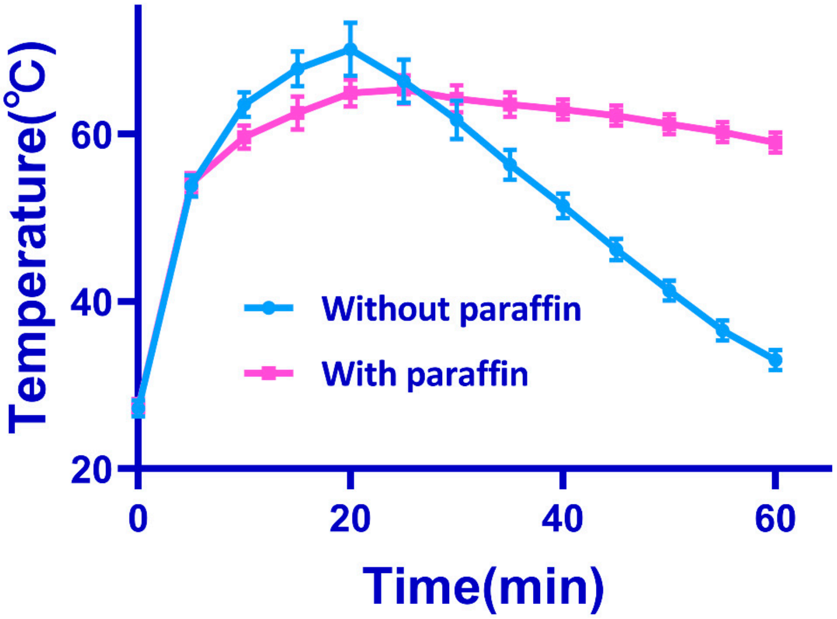

Figure S2. The temperature in the 3D-printed box with and without paraffin.

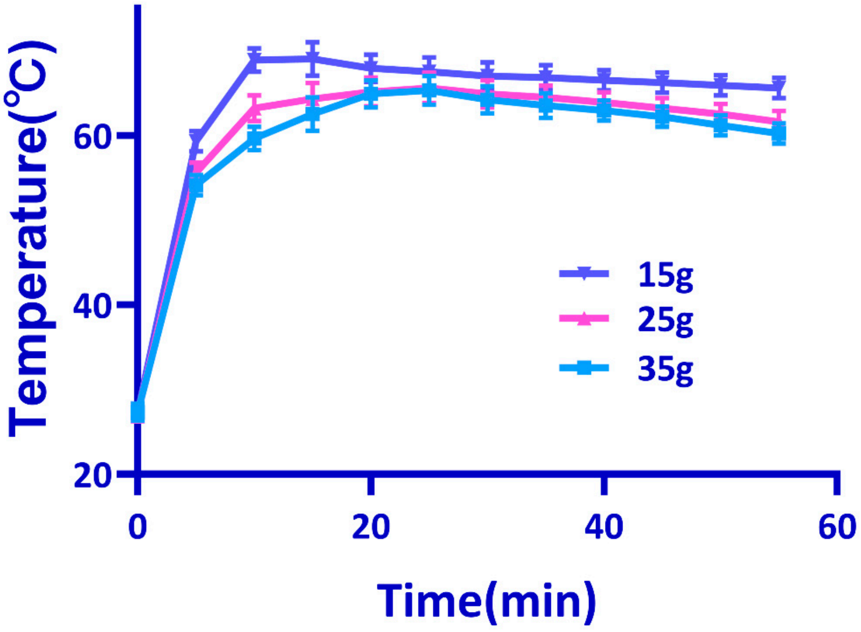

Figure S3. The temperature in the 3D-printed box with different masses of paraffin.

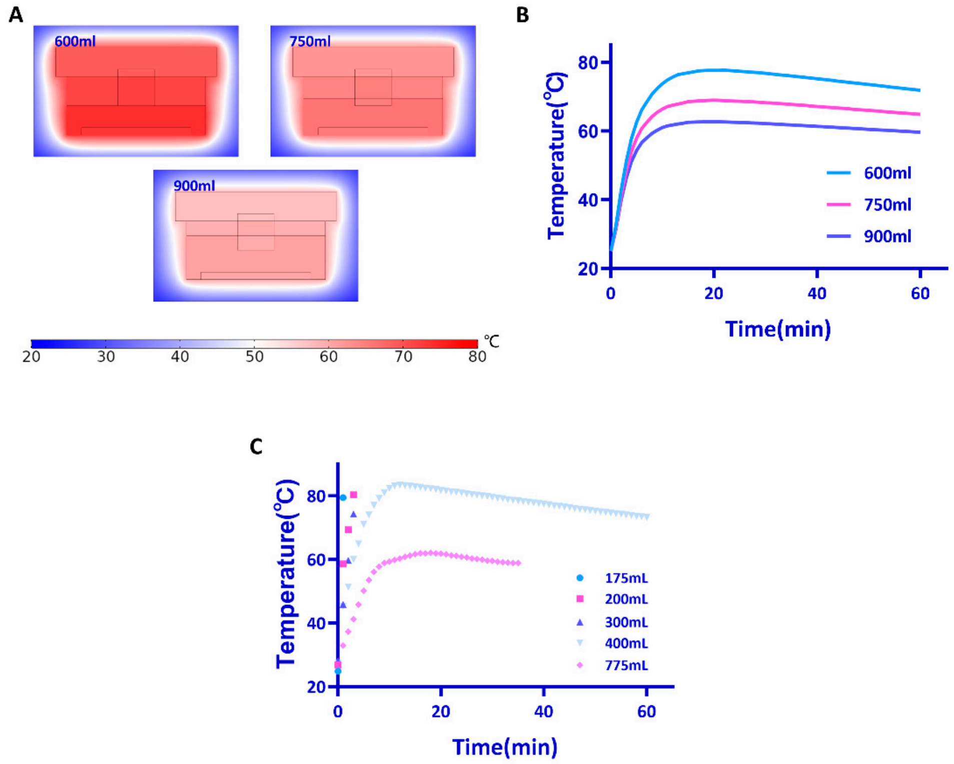

Figure S4. The temperature control effect with different amounts of water. A) Simulations of temperature distribution at 60 minutes, the water volume inside the foam box was 600ml, 750ml, and 900ml. B) Calculated temperature of adding different amounts of water. C) The temperature with different amounts of water. No further measurement after the temperature exceeds the RT-LAMP reaction temperature range.

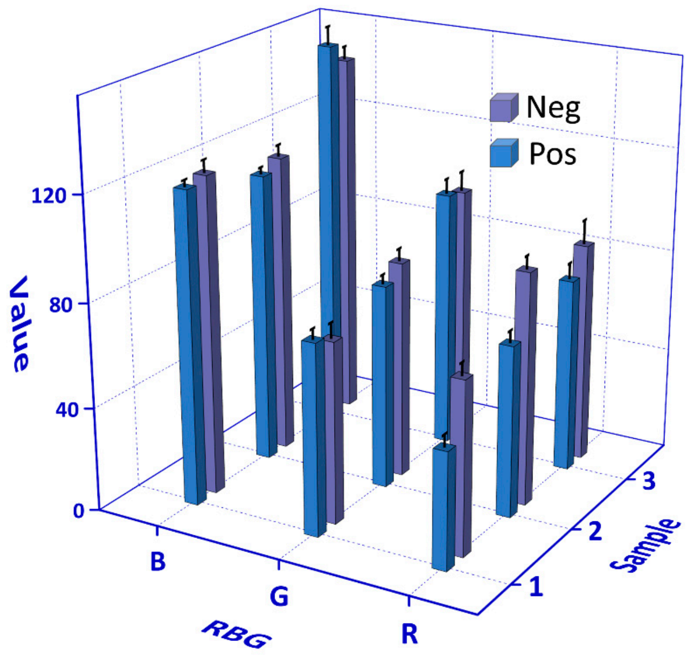

Figure S5. The RGB values of negative and positive (Sample 1: 4× 10^5 copies/µL of the SARS-CoV2 N gene plasmid, Sample 2: 4× 10^3 copies/µL of the SARS-CoV2 N gene plasmid, Sample 3: RNAs extracted from SARS-CoV-2 transfected cells) samples from three different tests.

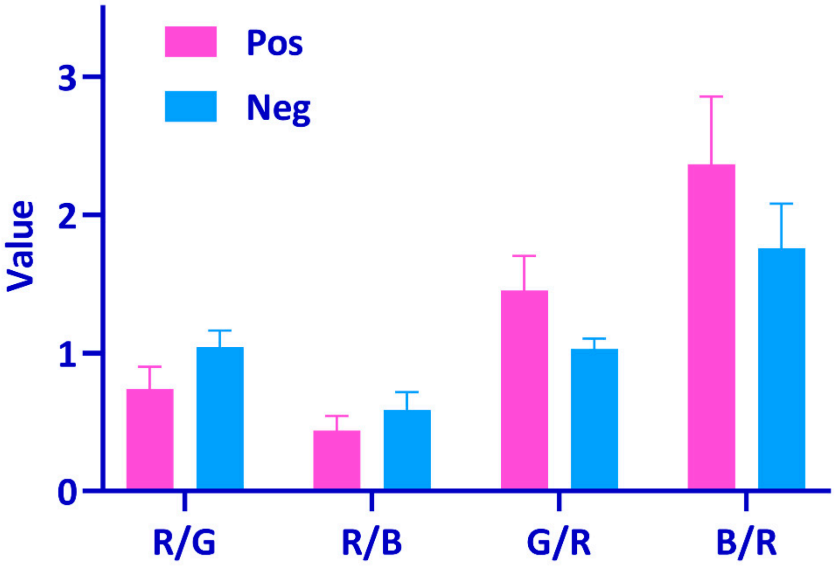

Figure S6. Analysis of the four possible RGB ratiometric combinations for positive and negative RT-LAMP samples.

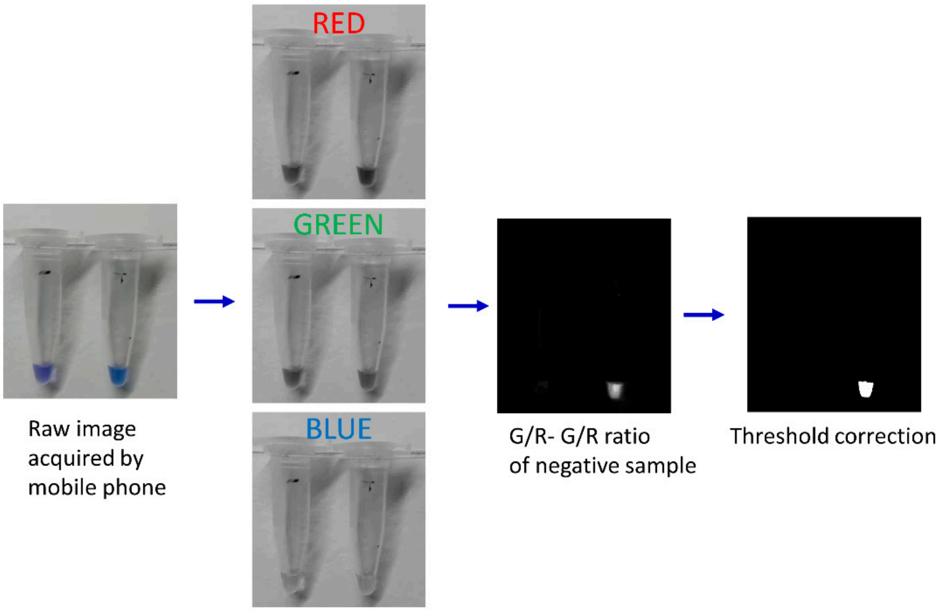

Figure S7. Each step of the G/R ratiometric approach for the self-testing system.

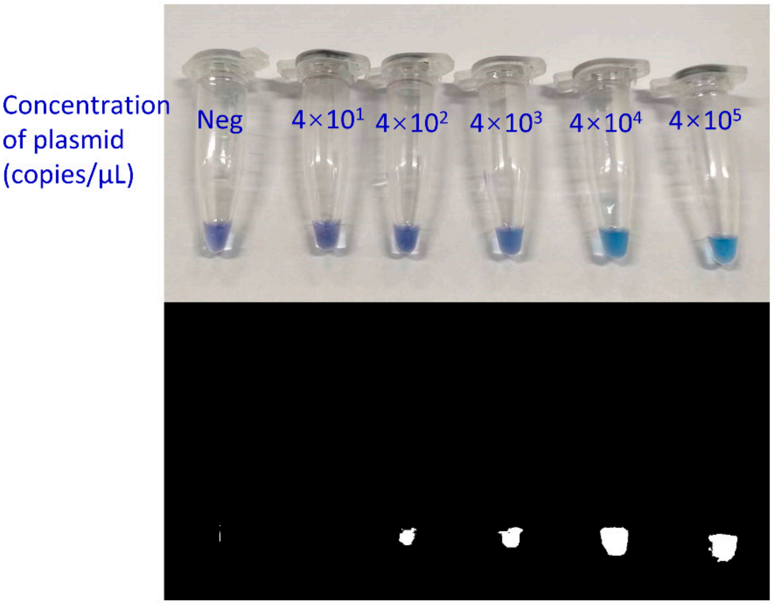

Figure S8. Visual readout of different concentrations of the plasmid. The top image is the cellphone original image. The bottom image is the binary image after G/R processing.

Table S1. Calculation of the cost of the self-testing system.

Reagents		Cost per device ($)	
**Reagents for per test**	**Primers (Ordered from Sangon BioTech)**	0.03	0.75
	Bst 2.0 Polymerase (New England Biolabs)	0.24	
	Bst 3.0 Polymerase (New England Biolabs)	0.12	
	dNTPs (Sangon Biotech)	0.02	
	Hydroxy naphthol blue (Aladdin)	0.01	
	$MgSO_4$ (New England Biolabs)	0.01	
	Isothermal Amplification Buffer (New England Biolabs)	0.02	
	extraction reagents (Sangon Biotech)	0.3	
**Other material**	3D-printed box fabricated by PLA (Ender-PLA 1.75mm)	0.39	0.85
	Foam box (Purchase from Taobao)	0.3	
	No. 64 Paraffin (Purchase from Taobao)	0.01	
	Swab (Purchase from Taobao)	0.04	
	Heating bag (Purchase from Taobao)	0.07	
	Tubes (Sangon Biotech)	0.04	
**Total**			**1.6**

(Calculated from retail cost in China)

Table S2. Reference primers were used in the study.

Primer	Sequence (5'-3')
FIP	GCGGCCAATGTTTGTAATCAGTAGACGTGGTCCAGAACAA
BIP	TCAGCGTTCTTCGGAATGTCGCTGTGTAGGTCAACCACG
F3	GCTGCTGAGGCTTCTAAG
B3	GCGTCAATATGCTTATTCAGC
LF	CCTTGTCTGATTAGTTCCTGGT
LB	TGGCATGGAAGTCACACC

Table S3. Optimized conditions of the self-testing system.

	Reagent	
**Reagents for optimized 20 µL RT-LAMP mixture.**	10 ×LAMP buffer	2µL
	8000 U/ml Bst 2.0 DNA polymerase	0.8µL
	8000 U/ml Bst 3.0 DNA polymerase	0.4µL
	Primer mixture	2µL
	25Mm dNTP	1.2µL
	3mM Hydroxy-naphthol-blue (HNB)	1µL
	100 mM $MgSO_4$	1.5µL
	Sample	1µL
	H_2O	10.1µL
**Reagents for optimized temperature control system**	Heating bag	50g
	Paraffin	25g
	Water added in the heater case	700ml

Table S4. Comparison with Lucira COVID-19 Test kit.

	This work	Lucira COVID-19 Test kit
**Detection method**	RT-LAMP	RT-LAMP
**Cost of per test**	1.6 $	29 $*
**Amplification time**	40 min	⩽30min*
**Throughput**	Maximum of 9 sample	1 sample

* Based on Lucira official website.
